# Role of Conformational Motions in Enzyme Function: Selected Methodologies and Case Studies

**DOI:** 10.3390/catal6060081

**Published:** 2016-05-27

**Authors:** Chitra Narayanan, David N. Bernard, Nicolas Doucet

**Affiliations:** 1 INRS—Institut Armand-Frappier, Université du Québec, 531 Boul. des Prairies, Laval, QC H7V 1B7, Canada (C.N.); 2 PROTEO, the Québec Network for Research on Protein Function, Engineering, and Applications, 1045 Avenue de la Médecine, Université Laval, Québec, QC G1V 0A6, Canada; 3 GRASP, the Groupe de Recherche Axé sur la Structure des Protéines, 3649 Promenade Sir William Osler, McGill University, Montréal, QC H3G 0B1, Canada

**Keywords:** enzyme catalysis, conformational dynamics, nuclear magnetic resonance, molecular dynamics simulations, HIV-1 protease, DNA polymerase β

## Abstract

It is now common knowledge that enzymes are mobile entities relying on complex atomic-scale dynamics and coordinated conformational events for proper ligand recognition and catalysis. However, the exact role of protein dynamics in enzyme function remains either poorly understood or difficult to interpret. This mini-review intends to reconcile biophysical observations and biological significance by first describing a number of common experimental and computational methodologies employed to characterize atomic-scale residue motions on various timescales in enzymes, and second by illustrating how the knowledge of these motions can be used to describe the functional behavior of enzymes and even act upon it. Two biologically relevant examples will be highlighted, namely the HIV-1 protease and DNA polymerase β enzyme systems.

## 1. Introduction

Proteins and enzymes are essential components of living cells. Among a variety of other functions, they act as hormones in cell signaling, protein transporters, antibodies in host defense, *etc.* Concomitantly, enzymes are some of the most efficient catalysts known to date, enhancing biochemical reaction rates up to 19 orders of magnitude relative to uncatalyzed reactions [1,2]. However, factors contributing to these large rate enhancements in enzyme-catalyzed reactions remain largely uncharacterized. The original structure-function paradigm, popular for many decades, portrayed enzymes as rigid structures with shapes that facilitate substrates, ligand and/or cofactor binding. This original model evolved over time, and theories were developed that led to the now-accepted induced fit, conformational selection, and transition-state stabilization models to explain the behavior of protein-ligand recognition and catalysis in the molecular function of enzymes [3]. Increasing evidence suggests that proteins sample a variety of distinct conformations (or sub-states) enabled by concerted atomic-scale dynamical fluctuations occurring over a wide range of timescales and acting on the primary, secondary, tertiary, and quaternary organization of their molecular structure [4]. Conformational transitions between highly and rarely populated states have been shown to play important roles in substrate recognition, binding, and product release, among others [5–8]. Advances in experimental and computational methodologies continue to offer new insights into enzyme conformational motions over functionally relevant timescales [9–12]. Interestingly, experimental [13,14], computational [15,16], and sequence-based [17,18] approaches have also revealed functional networks of concerted residue motions distant from the active site in selected enzyme systems. Correlations between the timescale of conformational fluctuations and that of catalytic turnover have been well established in a variety of enzyme systems, including cyclophilin A [14], RNase A [5,19,20], triosephosphate isomerase [21], and HIV-1 protease [22], among others. Further, the rate of conformational exchange has been shown to coincide with the rate-limiting step, such as product release, in some of these systems [19,20]. Conformational exchange between sub-states allows enzymes to sample higher energy conformations with structural and dynamical properties important for function such as ligand binding and allosteric regulation [11,16,23]. In addition to the millisecond conformational exchange, dynamics on faster timescales modulate the chemical environment through rearrangements in the active site, thus affecting enzyme function [24]. Taken together, a view is emerging whereby conformational fluctuations occurring over a range of timescales can affect enzyme function through conformational sampling along preferred pathways.

Over the past few years, a number of controversial statements relating ‘catalysis’ with ‘dynamics’ have been published in the literature, often giving rise to heated debates between experimentalists and theoreticians [25,26]. As recently outlined, these debates are often semantic in nature and can be traced back to actual definitions (and research field perceptions) of what ‘protein dynamics’ represents, in addition to which atomic-scale events are being observed and/or over which timescales they occur during enzyme-catalyzed reactions [25]. In the current report, we consider motions affecting enzyme function in a broad sense, *i.e.*, “any motion that, if impeded, would reduce the ability of the enzyme to function by the mechanism that it has evolved to execute” [27]. As such, we take into account motions on a wide range of timescales, from the fast, local picosecond-nanosecond (ps-ns) fluctuations of individual residues to the slower microsecond-millisecond-second (μs-ms-s) global conformational exchange experienced by enzymes. Conserved motional events occurring on a broad range of timescales could be involved in many and/or a selected subset of events occurring along the catalytic cycle of an enzyme, including ligand binding, recognition, substrate discrimination, structural rearrangements leading to the transition state, product release, *etc.* [25]. To clarify and prevent confusion, we never imply that ‘motions contributing to catalysis’ exclusively describe the fast femtosecond atomic motions involved in transition-state chemistry.

In the current account, we draw attention to a number of experimental and computational methodologies that have recently improved our understanding of catalytic and functional properties in two enzyme systems that closely rely on conformational dynamics for proper biological function. We do not pretend to cover the overwhelming number of theoretical approaches, methodologies, or enzymatic systems that previously illustrated the role of protein dynamics in enzyme function, but instead focus on recent reports where the combination of experimental NMR and computational techniques have emphasized the role of conformational exchange in inhibitor binding (HIV-1 protease) and fidelity in DNA repair through the selection of correct nucleotides (DNA polymerase β).

## 2. Selected Experimental and Computational Approaches for Sampling Conformational Motions in Proteins

**Figure 1:**
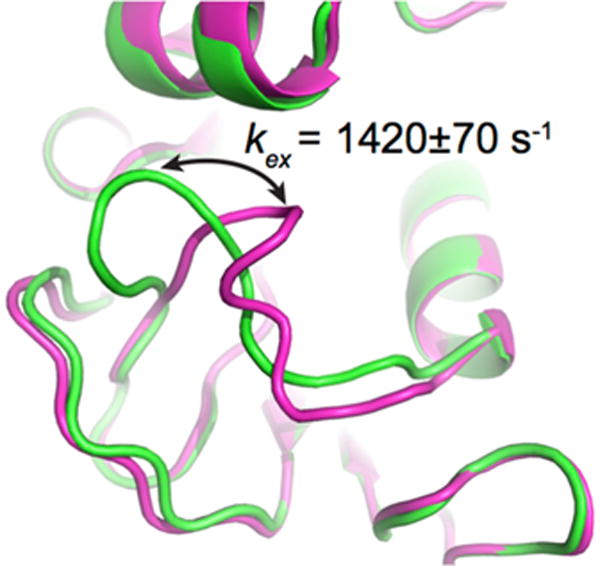
Conformational exchange experienced by the Met20 loop in *E. coli* DHFR, as probed by ^15^N-CPMG NMR relaxation dispersion experiments. The CPMG experiment is particularly well suited to extract exchange rates (*k*_ex_) between two or more conformations in solution on the timescale of catalysis (ms) in many enzyme systems, offering a measure of comparison between conformational exchange experienced by the enzyme and its catalytic rate (*k*_cat_). This method can also provide quantitative information on low-populated “invisible” excited sub-states and theft populations in solution (*p*_A_ and *p*_B_). The cartoon representation illustrates a simplified view of the closed (green) and occluded (magenta) conformations sampled by the Met20 loop in *E. coli* DHFR as it catalyzes hydride transfer, an atomic-scale movement essential to bacterial DHFR function and correlated with substrate/cofactor recognition and turnover in this enzyme (reviewed in [28,35,40]). Depicted PDB structures are 1RX2 (green) and 1RX7 (magenta).

A variety of approaches, including (but not limited to) NMR spectroscopy, X-ray diffraction, single molecule FRET, and computational simulations, have been used to probe conformational dynamics in proteins over nanosecond to millisecond timescales, offering the means to extract motions potentially relevant to biological function [4,28]. One of the most commonly used experiments to characterize local and global conformational events experienced by enzymes on the timescale of their catalytic rate *k*_cat_—typically occurring on the order of milliseconds—is the NMR Carr-Purcell-Meiboom-Gill (CPMG) relaxation dispersion experiment [29–33]. This methodology garnered considerable attention in recent years since motions experienced by enzymes on this physiologically relevant timescale can improve our understanding of catalysis and allostery, as the flexibility events uncovered can be analyzed in light of catalytic events. The CPMG method enables the detection of atomic-scale millisecond dynamics of enzymes in solution, either in the free form or bound to biologically significant ligands/analogues that mimic intermediate states along the chemical reaction [13,34,35]. On this time frame, molecular flexibility may involve the rearrangement of single residues and/or entire secondary structures moving in a coordinated fashion (for reviews see [9,36–38]) (Figure 1). In its typical form, the method involves the characterization of an equilibrium exchange process experienced by the N–H amide bond of amino acid residues, performed by recording the transverse relaxation rate constant (*R*_2_) as a function of *v*_CPMG_, the frequency with which refocusing pulses are applied in a CPMG pulse train [30,39]. These refocusing pulses are effectively invisible to amino acid residues that do not undergo conformational exchange in the protein, but they add an additional contribution (*R_ex_*) to *R*_2_ for exchanging residues—those experiencing motions on this millisecond time frame—and thus causing a decrease in NMR resonance intensity. Plotting this decrease in signal intensity as a function of *v*_CPMG_ not only allows the qualitative identification of residues experiencing conformational exchange in the enzyme, but fitting the curves to relaxation dispersion equations also allows the quantitative extraction of structural information related to the excited (less populated) sub-states (differences in chemical shifts, Δ*ω*), in addition to their population dynamics in solution (conformational exchange rates, *k*_ex_, and equilibrium populations, *p*_A_ and *p*_B_) (see [39] and references therein).

With regards to the timescale of protein motions, NMR is not limited to the μs-ms range. To this day, the model-free formalism developed by Lipari and Szabo in the early 1980s remains one of the preferred approaches to determine the atomic-scale dynamics of a protein in solution [41,42]. Using spin relaxation NMR data, the method primarily provides information on residue dynamics occurring on the ps-ns timescale, which are most often attributed to single residue side-chain rotations or local residue motions. The method measures spin relaxation rates for each residue amide N–H pair to extract order parameters (*S*^2^), conformational exchange parameters (*R*_ex_), and the correlation time (τ_c_), offering an interpretation of the internal amplitude of the N–H bond vector in solution [43–45]. The order parameter *S*^2^ provides an easily interpretable measure of the rigidity of each protein residue, where the *S*^2^ of a completely unrestricted N–H bond would theoretically correspond to a value of 0, while that of a fully rigid residue would be 1. For a number of residues that do not fit to simple calculation models, an *R*_ex_ exchange parameter can be estimated to achieve an adequate fit to the model-free analysis [45].

While the aforementioned NMR relaxation analyses provide detailed information on the molecular flexibility experienced by enzymes in solution, other experimental methodologies offer additional details pertaining to potentially significant biological motions. Single-molecule Förster Resonance Energy Transfer (single-molecule FRET) [4,46] and site-directed spin labeling Double Electron-Electron Resonance (DEER) spectroscopy [47]—also called pulsed Electron Paramagnetic Resonance (pulsed-EPR)—are both used to determine the distance between two labels, ranging up to a few tens of Ǻ. In DEER, a pair of cysteine residues is chemically modified with electronic spin labels, namely the nitroxide radical of a methylthiosulfonate moiety [22,48]. The magnitude of the dipolar coupling between the two spins can be extracted from the measured DEER echo curves, and this magnitude is inversely proportional to the cube of the distance between the two spins [49], thus producing a distance profile. Populations of the different states of the protein can then be evaluated by deconvoluting the distance profile. Much like with protein crystallography, a disadvantage of the technique is the requirement of cryogenic temperatures [47], which does not provide an accurate description of biological conditions. On the other hand, single-molecule FRET can be used in solution and at physiologically relevant temperatures, much like NMR. This technique also requires labeling of two distinct sites on a protein, this time using two different fluorophores. Since these fluorophores are selected so that the absorption spectrum of the first (the acceptor) matches the emission spectrum of the second (the donor), the fluorescence energy can be transmitted from the donor to the acceptor if they are in close proximity to each other. Thus, the emission wavelength of the donor will disappear and the emission wavelength of the acceptor will be detected as a function of distance. By measuring the FRET efficiency, the inter-fluorophore distance can be calculated. The single-molecule version of this experiment can thus allow one to witness whole domain reorientations in a protein. This technique also has the advantage of being performed in real-time [4,46].

While most experimental methodologies yield quantitative information on flexibility events, they often fail to provide a clear visual representation of the actual atomic-scale motions experienced by an enzyme in solution [44]. In contrast, computational molecular dynamics (MD) simulations trace the position and interaction of atoms over time based on classical mechanics principles, to provide an atomic-scale time evolution of conformational properties in proteins and other biomolecules. Until recently, MD simulations typically sampled over hundreds of nanoseconds, while protein motions span a much wider range of timescales [4], with many catalytically relevant motions occurring on the same timescale as the catalytic constant *k*_cat_, *i.e.*, typically milliseconds or slower [19,25,50,51]. This presents an existing limitation of computational MD simulations. Advances in software and hardware capabilities have facilitated access to longer timescales, with several recent studies reporting ms timescale simulations [52–54]. While a single ms trajectory has been achieved using Anton [55], a specialized supercomputer for protein simulations, access to the ms timescale with traditional, unbiased MD simulations still remains limited.

A variety of enhanced sampling techniques such as accelerated MD (aMD) [56,57], umbrella sampling [58], steered MD [59], and metadynamics [60–62], among others, have been developed to improve sampling of the conformational landscape of proteins. In aMD, the potential energy landscape is modified by raising energy minima that are below a threshold, therefore minimizing the energy barriers separating states, and facilitating access to conformational states not easily accessible through traditional MD [57]. This approach has been used to characterize dynamics on the μs-ms timescale in a variety of protein systems [63]. A combination of normal and accelerated MD simulations was recently used to characterize the role of conformational dynamics in stabilizing the transition state in the enzyme cyclophilin A [64]. Umbrella sampling uses a biasing potential to characterize the higher energy states along a reaction coordinate [65]. Metadynamics involves the use of a history-dependent biasing potential that acts on collective variables corresponding to select degrees of freedom [60]. A combination of NMR and metadynamics was recently used to characterize the conformational ensemble and the free energy landscape of the highly dynamic helix 1 of the prion protein [66]. While these approaches have clear benefits from enhanced sampling, the requirement of the knowledge of start and end structures (umbrella sampling), or the selection of suitable reaction coordinates (metadynamics), among others, limit their application [67].

Other advanced sampling methods, such as the Markov State Model (MSM, also known as the kinetic network model) [68–70] and Quasi Anharmonic Analysis (QAA) [11,71], facilitate the identification of conformational sub-states, equilibrium populations of sub-states, and transition probabilities between these sub-states. MSMs are constructed from trajectories obtained from MD simulations. In these models, the conformational space is discretized into multiple states, and a network connecting these states is created from the MD simulations. A transition matrix corresponding to the probability of transitions between different states is constructed to characterize the kinetics of transition between states. MSMs have been applied to sample a wide range of timescales associated with protein folding, functional dynamics, ligand binding and for characterizing intrinsically disordered proteins [72–74]. QAA uses higher order statistics to describe positional fluctuations and the coupling of these fluctuations in different regions of the protein [71]. It allows the characterization of conformational sub-states along a reaction pathway and the identification of sub-states with structural and dynamical properties important for function [75]. This approach was used to identify the hierarchical organization of conformational sub-states in ubiquitin and the T4 lysozyme, and the conformational sub-states associated with the cis/trans isomerization catalyzed by cyclophilin A were also characterized [75]. This technique was recently used to characterize conformational sub-states associated with the interconversion between reactant and product states in wild-type and mutant forms of RNase A [76]. Recent years have also seen advances in the integration of experimental and computational approaches for characterizing the dynamics of proteins. NMR chemical shifts and residual dipolar couplings have been used as replica-averaged structural restraints to characterize the conformational fluctuations in RNase A and the hen lysozyme, respectively [77–79].

## 3. Case Studies

### 3.1. HIV Protease

The protease of the type 1 human immunodeficiency virus (HIV-1 PR) is an aspartyl proteinase that cleaves the polyprotein product of the *gag-pro-pol* gene and the Gag protein itself, both of which are vital steps in the maturation of the virus [80]. The three most prevalent subtypes of the virus are those whose proteases have been the most studied: subtypes B, C, and CRF_01 A/E, which are the prevalent HIV subtypes in North America and Europe, Africa, and Asia, respectively. Even though all three subtypes have a distinct wild-type (WT) protease, subtype B is the most widely characterized [47]. To date, nine Food and Drug Administration (FDA)-approved inhibitors targeting this protein have been used to treat HIV/acquired immune deficiency syndrome (AIDS)-infected patients. However, multidrug resistance to protease inhibitors has sparked renewed interests in uncovering distinct inhibitors targeting HIV-1 PR, and thus understanding drug resistance acquisition mechanisms is of utmost importance [81].

**Figure 2:**
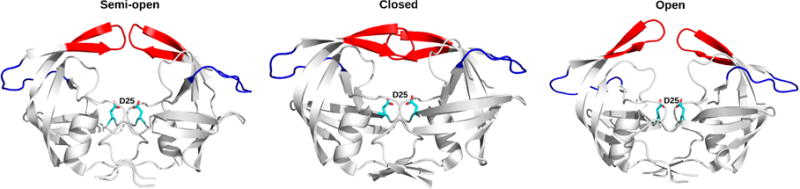
Conformational states experienced by HIV-1 PR as it proceeds through its catalytic cycle. Crystal structures of HIV-1 PR in the semi-open (PDB 1HHR), closed (PDB 1HVR) and wide open (PDB 1TW7) conformations. The catalytic Asp25 residues from each protomer are depicted as cyan sticks and the “flaps” are depicted in red. The hinge loop, corresponding to residues 34–42, is shown in blue. The curled/tucked conformation of HIV-1 PR is not depicted, since it has never been crystallographically resolved.

HIV-1 PR is only active in its homodimeric form. The 99-amino-acid protomers assemble in a symmetric configuration, forming a tunnel. The protomers each provide one of the catalytic aspartate residues (Asp25, located in the tunnel), while access to the catalytic site is allowed through the opening and closing of a pair of β-hairpins, termed “flaps” (residues 43–58) (colored red in Figure 2). The protomers interact with each other through their *N*- and *C*-termini, as well as at the tip of the flaps [82]. A previous report briefly discusses the atomic-scale dynamics experienced by WT HIV-1 PR, describing how movements of the flaps originating from the isomerization of Gly51 are transmitted to the active site [83]. This vital conformational transition, identified as early as 1995, involves the 180° reorientation of the Gly51 backbone N–H vector, exchanging between β-turn types I and II, a process taking place over a 10 μs timescale, as determined from *R_ex_* values extracted using the Lipari-Szabo model-free formalism mentioned above [84]. This unique residue reorganization represents the sole significant structural difference between otherwise symmetrical protomers in the crystal structures. Most commonly, Gly51 is found in the *L*-conformation in one protomer and in the *D*-conformation in the opposing protomer [22]. Model-free analyses also confirmed that the flap region, along with the hinge loop (residues 34–42), are the primary regions in which sub-nanosecond timescale residue dynamics are localized [85].

NMR relaxation and computational MD simulations demonstrated that flaps can adopt a variety or conformations as the enzyme proceeds through its catalytic cycle [47,86,87]. These flaps alternate between a “closed” conformation, a “semi-open” conformation, a “wide open” conformation, and a “curled/tucked” conformation (Figure 2). The flaps close over the active site to maintain the substrate inside the catalytic pocket and to provide a gating mechanism for substrate binding, but they also interact with each other in the absence of substrate or inhibitor [85]. A combination of DEER measurements, NMR relaxation experiments, and MD simulations provided information on Asp25 and flap dynamics in each protomer during catalytic turnover. The μs-ms timescale motions of Asp25 and Gly27 were found to correlate with those of the flaps [22]. It was also observed that the more flexible *L*-Gly51 must be located on the same protomer as the protonated general acid Asp25-COOH for efficient catalysis to occur, while the more rigid *D*-Gly51 must be located on the protomer bearing the unprotonated general base Asp25-COO^−^. The rate-limiting step of catalytic turnover was shown to be linked to isomerization of the flap tips towards the type I and type II β-turn configurations [22], further illustrating the functional importance of conformational exchange in this enzyme system.

The kinetic network model, constructed by discretizing the conformational space of atomistic simulations into a series of related sub-states, was used to determine the transitions between the semi-open, closed, and open conformations of HIV-1 PR [88]. This approach facilitates the characterization of interconverting rates between kinetically relevant sub-states, and can predict kinetic quantities on slower (millisecond) timescales [72,89]. Deng *et al*. combined replica exchange molecular dynamics simulations with transition path theory to characterize the diversity and temperature-dependence of kinetic pathways between the three conformational states of HIV-1 PR. They showed that transitions between semi-open and closed states occur on a fast timescale (~33 ns), while transitions between semi-open and open states were infrequent (~375 ns), consistent with NMR observations showing motions on the fast sub-nanosecond and slower microsecond timescales [88].

Not all studies agree on which conformation is the most likely populated form of the apo WT protease in solution. Studies using the DEER method concluded that the semi-open conformation is the most populated state, independent of the subtype [22,47], while NMR residual dipolar coupling (RDC) measurements suggested that the closed conformation was predominant [90]. However, all studies agree that the populations of these conformers change upon inhibitor binding, with the most potent inhibitors favoring the “closed” conformation [47,91–93]. In all common subtypes of WT HIV-1 PR (subtypes B, C, and CRF_01 A/E), the residence time of inhibitors in the active-site pocket, and thus the inhibitory strength, is correlated with the degree to which said inhibitor induces flap closure. Consequently, many drug resistance-inducing mutations perturb the dynamic behavior of the flaps relative to the WT enzyme [47]. Different inhibitors cause varying population shifts among the four possible flap conformations. According to DEER measurements, while inhibitors indinavir, nelfinavir, and atazanavir trigger very small differences in populations in all three subtypes—the semi-open conformation being the most populated—all other six FDA-approved inhibitors strongly shift the population equilibrium towards the closed configuration. Interestingly, the same six inhibitors also make the curled/tucked conformer much more likely in subtype CRF_01 A/E [47]. While smaller in scope than those of the flaps, conformational changes in the hydrophobic core predicted by MD simulations were also confirmed to impact protease activity [94]. In the WT HIV-1 PR, the two halves of the hydrophobic core slide over each other during these rearrangements, and cross-linking these two halves—thus impeding hydrophobic core rearrangements—causes a 150-fold reduction in catalytic efficiency [94,95]. Also, mutations distal to the active site in the hydrophobic core are hypothesized to cause drug resistance by altering the flexibility of the protein [94].

**Figure 3:**
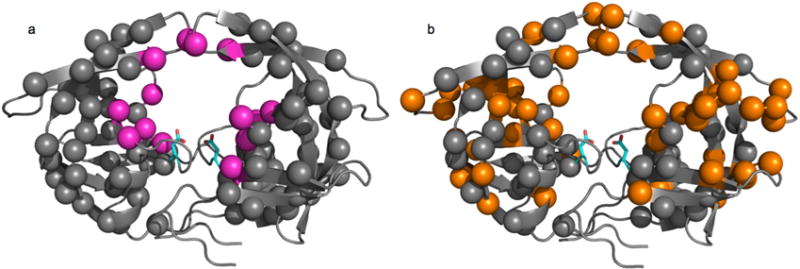
Location of drug resistance and conformational mutations in HIV-1 PR. Structural mapping of various mutations on the crystal structure of apo HIV-1 PR (1HHR). The catalytic Asp25 residue is depicted as cyan sticks on each enzyme protomer. All spheres represent a residue documented to undergo mutation in HIV-1 PR [97]. (**a**) Location of drug resistance-inducing mutations. Magenta spheres represent mutations that impair inhibitor binding through direct active-site contacts (primary mutations) and gray spheres represent distal indirect mutations (secondary mutations); (**b**) Location of mutations documented to affect the conformational sampling of HIV-1 PR, depicted as orange spheres. Gray spheres are other drug resistance-inducing mutations. All mutations were compiled from references listed in the text.

Previous reports have highlighted that many distinct mutations can arise in patients developing drug resistance to HIV treatment [96]. Some of these mutations are located in the active-site pocket and directly impair inhibitor binding (primary mutations), while others are more distal and indirectly induce resistance (secondary mutations) (Figure 3). None of these distal mutations cause significant changes in either *k*_cat_ or *K*_M_ in HIV-1 PR, suggesting that conformational events experienced by the enzyme play a significant role in resistance acquisition, which was confirmed by NMR, MD simulations, and DEER spectroscopy. Mutations in 45 of the 99 residues in HIV-1 PR have been linked to drug resistance *in vitro* [97], and a quarter of the residues have been linked to drug resistance in patients, arising through evolutionary selective pressure driven by protease inhibitor therapy. However, mutations at only seven sites can be linked to direct active-site modifications (Figure 3a) [92,97,98]. These primary mutations often decrease ligand binding affinity, both for inhibitors and natural substrates. Secondary mutations often arise to restore activity towards substrates, sometimes as a result of conformational behavior. For instance, incorporation of I15V, E35D, R41K, and R57K was demonstrated by DEER to increase backbone flexibility in WT HIV-1 PR [48]. These mutations, which are all believed to act as function-restoring secondary replacements, result in the increased population of the curled/tucked flap conformation, which does not normally occur in apo subtype B. Other secondary mutations can favor drug resistance. For example, 13 out of the 19 hydrophobic core residues are associated with drug resistance acquisition, despite the absence of direct contacts with inhibitors [94]. Importantly, the accumulation of mutations in HIV-1 PR does not necessarily lead to increased drug resistance [48,93,99]. Indeed, alterations in inhibitor-protein interactions caused by secondary mutations correlate only partly with *K_i_* variations. The explanation most often put forward is that these secondary mutations alter the dynamic ensemble of the protein, which propagates through the entire structure (Figure 3b), sometimes even affecting the dynamically constrained catalytic Asp25 residue [90,92,93,98]. Thus, if a secondary mutation does not alter protein dynamics, it will have no effect on inhibitor binding. Nonetheless, increased drug resistance is the usual outcome of mutational accumulation. For instance, a DEER study comparing a pediatric AIDS patient-derived HIV-1 PR before and after protease inhibitor treatment found that the accumulation of secondary mutations shifts the populations of the conformers to favor the open-like conformations, when it originally favored the closed conformation [100]. These authors emphasize the importance of the L63P replacement on the behavior of the protein in the pre-treatment sample, arguing that it likely affects hydrophobic core packing, and thus favoring the closed conformation in the apo protein.

These results illustrate how knowledge of the conformational transitions in HIV-1 PR may help develop new drug strategies against AIDS, further exemplifying the importance of concerted and organized molecular flexibility for the proper catalytic function in this enzyme. Targeting conformational transitions has already provided promising treatment alternatives for new HIV-1 PR drug leads. The use of MD to screen for new competitive or allosteric inhibitors that target MD-identified, transiently open surface cavities was shown to be effective against mutants of HIV-1 PR [92,101,102]. In one case. a designed allosteric inhibitor was demonstrated to be effective *in vitro* using enzymatic essays, even against drug-resistant variants [102]. Interestingly, the allosteric inhibitor strategy used in these cases was designed to lock a conformation in place, thus preventing the catalytic cycle from talking place.

### 3.2. DNA Polymerase β

**Figure 4:**
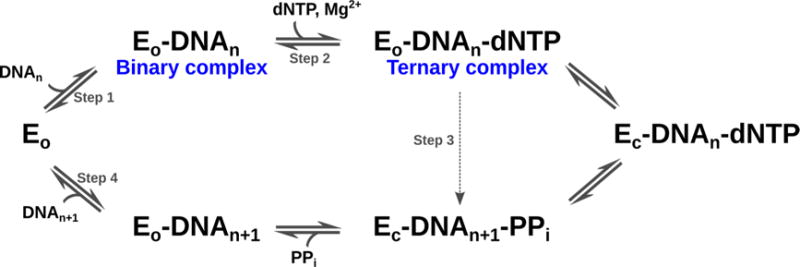
Schematic representation of the catalytic mechanism of Pol β. *E_o_* and *E_c_* correspond to the open and closed conformations of the enzyme. Sequential binding of the DNA (DNA*_n_*) and dNTP leads to the formation of the binary (step 1) and ternary complexes (step 2), respectively. Subsequent conformational rearrangement of the enzyme (*E_c_*) aligns the active site for catalysis (step 3). The chemical step of catalysis is followed by the release of pyrophosphate (PP*_i_*) and the DNA product (DNA*_n_*_+1_) (step 4). Figure adapted from [108,109].

DNA polymerase β (Pol β) is a 39 kDa monomeric enzyme involved in the base excision repair of damaged DNA. Pol β prefers short-gapped or single-base-gapped DNA substrates [103] and is responsible for the repair of around 20,000 DNA lesions per cell per day [104]. Mutations of some residues in Pol β have been shown to increase the error rate of the enzyme; further, overexpression of Pol β has been implicated in nearly 30% of human cancers [105,106]. Incorporation of correct nucleotides is an essential step in the catalytic mechanism of the enzyme. The structure of Pol β consists of two domains: an 8 kDa lyase domain involved in the removal of deoxyribose 5′-phosphate, and a 31 kDa polymerase domain which performs template-directed DNA synthesis [107]. The polymerase domain is further composed of nucleoside 5′-triphosphate selection, nucleotide transferase, and DNA-binding subdomains, corresponding to the fingers, palm, and thumb regions, respectively. The catalytic mechanism of Pol β, which requires two Mg^2+^ ions as cofactors, is shown in Figure 4.

**Figure 5:**
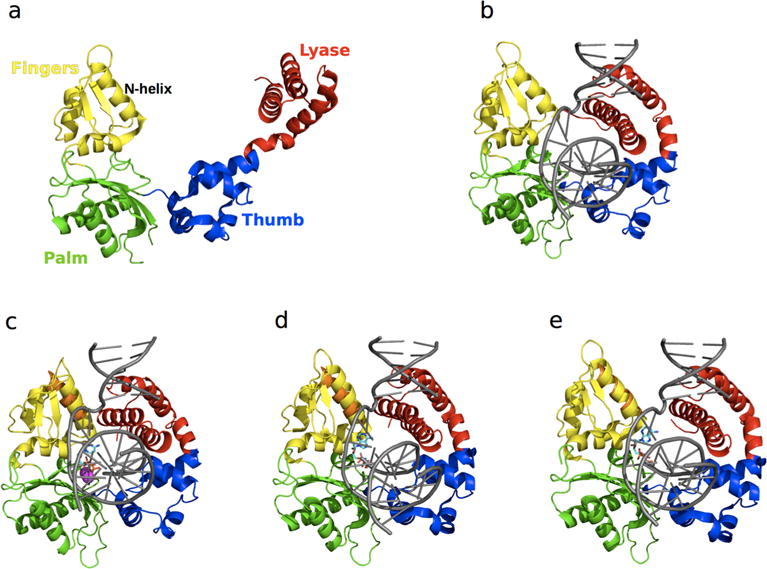
Structure of free and ligand-bound states of Pol β. The lyase domain is shown in red while the thumb, palm, and fingers subdomains of the polymerase domain are shown in blue, green and yellow, respectively. Pol β in the (**a**) apo (PDB 1BPD) form is characterized by an open conformation; (**b**) binary complex (PDB 1BPX) formation is accompanied by a 40 Ǻ conformational shift of the lyase domain to form *a* compact “open” conformation; (**c**) matched dNTP-bound ternary (PDB 1BPY) complex shows a 10 A shift of the N-helix of the fingers domain relative to the binary form (in orange), leading to the formation of a “closed” conformation. Binding of mismatched dNTPs has been shown to adopt (**d**) closed (PDB3C2M) and (**e**) open (PDB 4F5P) conformations of the N-helix. For oomparison, the *N*-helix from the binary form is shown in orange in the ternary complexes in **c–e**, illustrating subtle yet functionally significant conformational exchange in this enzyme. DNA substrates in all the binary and ternary forms are shown in grey (**b**–**e**), the dNTPs in **c**–**e** are shown as cyan sticks, and the Mg^2+^ ions are represented as magenta spheres in c.

**Figure 6:**
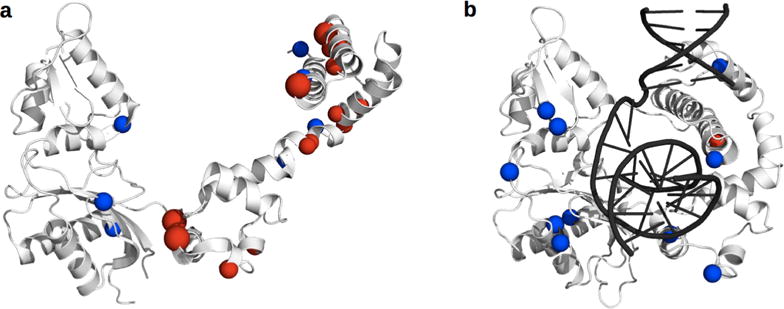
Conformational exchange experienced by Pol β. Residues displaying elevated *R*_2_ and ^15^N-CPMG relaxation dispersion curves are shown as blue and red spheres, respectively, corresponding to Cα atoms. (**a**) The apo form shows millisecond dynamics predominantly in the lyase domain (residues 1**–**90). Residues with *k_ex_* ~1400 s^−1^ are shown as smaller spheres while residues with *k_ex_* ~4000 s^−1^ are shown as larger red spheres. Few residues in the other polymerase domains show elevated *R*2 values; (**b**) Onlyresidue Glu21 displays ^15^N-CPMG relaxation dispersion in the binary form. Other residues displaying elevated *R*2 values are located in the polymerase domain of Pol β. DNA in the binary form is shown in black. The relaxation data used for preparing this figure were taken from reference [8].

Crystallographic studies have shown that Pol β undergoes large conformational changes upon substrate binding [110–115] and suggest an induced fit mechanism for the binding of correct nucleotides [111]. The apo form of the enzyme is characterized by an extended conformation of the lyase domain (Figure 5a), which adopts a compact open conformation upon DNA binding (Figure 5b). Binding of the nucleotide 5′-triphosphate (dNTP) promotes closing of the *N*-helix in the finger subdomain (Figure 5c). NMR studies were recently performed to characterize the millisecond timescale conformational exchange experienced by the apo and DNA-bound binary complex forms of Pol β [8]. The authors of that study showed that conformational exchange in the apo form occurs primarily in the lyase domain and the base of the thumb in the polymerase domain, and corresponds to residues that are either in direct contact or in close proximity to the substrate in the binary complex. In contrast, the number of residues displaying conformational exchange in the binary complex with DNA was significantly reduced. These residues are primarily located in the polymerase domain, suggesting that DNA binding limits the motions in the lyase domain (Figure 6). Chemical shift variations were located in the lyase domain, associated with large conformational changes experienced by Pol β upon DNA binding (Figure 5a,b). Comparison of the conformational exchange relaxation rates with chemical shift variations between the apo and binary forms indicates that the enzyme in the apo form samples conformations adopted by the DNA-bound binary form, thus contradicting the induced fit mechanism proposed by the aforementioned crystallography experiments, at least for the formation of the binary complex (step 1 in Figure 4). Interestingly, residues displaying conformational exchange in the apo and binary complex forms correspond to mutational sites known to affect Pol β activity and identified in colon cancer tumors [116]. Taken together, these results hint at the potential role of conformational motions in the catalytic function of Pol β.

Conformational changes in both Pol β and substrates have been suggested to play a critical role in the selection and incorporation of correct nucleotides [117]. Experimental [109,111–113,118] and computational [119,120] studies suggest an induced fit mechanism for the selection of correct nucleotides (step 2 in Figure 4), which form a Watson-Crick base pair with the DNA substrate. The mechanism involved in the selection of correct dNTP binding and discrimination is a hotly debated topic [109]. Crystal structures with open (Figure 5d), closed (Figure 5e), and intermediate conformations have been obtained with mismatched or incorrect dNTPs, such as dA that cannot form a correct base pair with dG of the DNA substrate. Previous fluorescence studies [118,121,122] showed movements in Pol β upon incorporation of dNTPs and cofactors, while more recent studies [108] indicate the rapid movement (ms timescale) of the fingers domain upon binding to correct dNTPs. Incorporation of incorrect dNTPs did not induce these conformational changes.

Moscato *et al.* characterized the effects of Pol β binding to correct and incorrect dNTPs on the millisecond timescale using NMR relaxation dispersion experiments [109]. Binding of correct dNTP to Pol β led to large chemical shift variations localized in the fingers subdomain, similar to previous observations [111,114]. No residue displayed conformational exchange, suggesting a stable conformation of the ternary complex on the millisecond timescale, similar to that observed for the binary complex. In contrast, chemical shift changes upon addition of the incorrect dNTP were much smaller than those observed for the correct dNTP. Comparison of the direction of chemical shift variations during titrations, performed using chemical shift perturbation [123], revealed that the conformation of the mismatching complex is not on-pathway to the closed conformation observed upon binding of the correct nucleotide. Further, binding of the mismatched dNTP led to enhanced millisecond dynamics of 10 residues with conformational exchange rates varying between 500 and 2700 s^−1^. These results support an induced fit mechanism for the ternary complex (step 2 in Figure 4), where binding of the correct nucleotide promotes a shift from the open binary complex form to a closed ternary complex conformation.

## 4. Concluding Remarks

The intrinsic dynamical properties of enzymes have been shown to impact the catalytic function in a variety of well-characterized systems. Advances in experimental and computational approaches continue to offer insights into the important role of conformational fluctuations in function on timescales relevant for enzyme catalysis and other biological events. Conformational exchange on the catalytically relevant timescale facilitates sampling of excited-state conformations or sub-states, aiding in various steps along the reaction pathway, such as substrate recognition, binding, and product release. In this review, we depicted the important effects of conformational dynamics on correct substrate selection and substrate/inhibitor recognition and binding in two enzyme systems. These observations also emphasize the role of dynamic fluctuations in tuning the conformational landscape for efficient catalysis, and present a potential approach for using dynamics to modulate function and for designing better inhibitors in drug design.
